# Cross-Sector Collaboration for a Healthy Living Environment—Which Strategies to Implement, Why, and in Which Context?

**DOI:** 10.3390/ijerph17176250

**Published:** 2020-08-27

**Authors:** Natascha J. E. van Vooren, Hanneke W. Drewes, Esther de Weger, Inge M. B. Bongers, Caroline A. Baan

**Affiliations:** 1 Department of Quality of Care and Health Economics, Centre for Nutrition, Prevention and Health Services, National Institute for Public Health and the Environment (RIVM), P.O. Box 1, 3720 BA Bilthoven, The Netherlands; Hanneke.drewes@rivm.nl (H.W.D.); Esther.de.weger@rivm.nl (E.d.W.); 2 Tranzo, Tilburg School of Social and Behavioural Sciences, Tilburg University, P.O. Box 90153, 5000 LE Tilburg, The Netherlands; Inge.bongers@ggze.nl (I.M.B.B.); Caroline.baan@rivm.nl (C.A.B.); 3 Mental Health Care Institute Eindhoven, de Kempen, P.O. Box 909, 5600 AX Eindhoven, The Netherlands; 4 National Institute for Public Health and the Environment (RIVM), P.O. Box 1, 3720 BA Bilthoven, The Netherlands

**Keywords:** cross-sector collaboration, healthy living environment, realist evaluation

## Abstract

Background: Working toward a healthy living environment requires organizations from different policy domains and nongovernment partners involved in public health and the living environment to collaborate across sectors. The aim of this study is to understand how this cross-sector collaboration for a healthy living environment can be achieved. Methods: The realist evaluation approach was used to investigate what strategies can be used in which contexts to achieve cross-sector collaboration. The “Collaborative Adaptive Health Networks” framework was used as a theoretical framework. Seventeen partners of three Dutch projects collaborating for a healthy living environment in different regions were interviewed about their experiences during the initiating phase of their projects. Results: Seven themes for achieving cross-sector collaboration were identified, namely creating a feeling of equivalence, building trust, bridging different perspectives, providing clarity regarding roles and tasks, creating commitment, creating active engagement, and understanding whom to engage and when. For each theme, the strategies that were used, and why, were specified. Conclusion: This study provides new insights in how cross-sector collaboration for a healthy living environment can be achieved in different contexts. Whether the start of a cross-sectoral collaboration is successful is largely influenced by the choice of leadership and the interorganizational relations.

## 1. Introduction

People’s health is influenced by a variety of factors, such as personal factors and lifestyle, and factors related to the physical and social environment [1]. Among these factors, the relationship between the physical environment and health has been studied extensively, highlighting the need to integrate the corresponding policy sectors and nongovernment partners to work together toward a healthy living environment [2,3]. The interest in integrating public health with other policy domains has increased over the years with programs such as Health in All Policies and the Whole-of-Government approach [4]. Based on studies that examined such programs, cross-sector collaboration was found to be of major importance when integrating public health with other sectors [5,6]. However, cross-sector collaboration is also found to be a challenge due to stakeholders having different perspectives, norms, and values [4,7,8], especially when integrating the domains of health and the physical environment [9]. Therefore, a better understanding of how partners from different sectors can successfully collaborate for a healthy living environment is required.

Cross-sector collaboration for health has been studied widely. (Multiple) Case studies provided insight into facilitators and barriers for cross-sector collaboration for health by reflecting on collaboration for health partnerships and collaboration for developing health in all policies (e.g., [10,11,12,13,14,15]). In these studies, facilitators such as creating a mutual aim and objectives, building trusting relationships, and having sufficient communication and barriers such as having limited (financial) resources and having different professional languages and values are found to be of importance for the success of cross-sector collaboration for health [10,11,12,13,14,15]. Studies that look at cross-sector collaboration for health from a more general and theoretical perspective align with the multiple case studies on the factors that are of relevance for collaboration for health [8,13,16,17,18]. In addition these studies acknowledge the complexity of cross-sector collaboration, including the need to understand the influence of the context on the success and outcomes of the collaboration [8,19]. An in-depth understanding of the influence of the local context on collaboration for health is however limited. One of the methodological approaches that can be used to examine the effect of context on cross-sector collaboration for health is the realist evaluation (RE) approach [20].

The realist evaluation approach enables the deeper understanding of what works, for whom, how, and in which context. RE involves the search for causal relations between contexts, underlying mechanisms and their outcomes, when certain intervention resources or strategies are implemented [20]. This approach was found useful in previous studies aiming to evaluate complex programs for integrating public health with others sector, such as Healthy Cities [21] and Health in All Policies [22]. However these were not yet specifically targeted at cross-sector collaboration for a healthy living environment.

From 2019 onward, the Dutch National Institute for Public Health and the Environment (RIVM) has been a partner in multiple projects with different local and regional partners such as professionals from public health organizations, municipalities, provinces, and citizens aiming to collaborate for a healthy living environment. The aim behind the projects was to tackle problems regarding public health issues and the physical environment. These issues included, for example, concerns regarding local air quality, providing a knowledge base regarding the effects of climate change on health, and including citizens in policy priority setting for a healthy living environment. The initial development of cross-sector collaboration within these projects provided an opportunity to gain more understanding in the collaboration processes for a healthy living environment. This study investigated partners’ experiences with the initiating phase of the projects. The further development of these projects will be evaluated in the coming years. This study is focused on understanding how cross-sector collaboration for a healthy living environment can be achieved, by uncovering what strategies have been implemented, why, and in which contexts, for cross-sector collaboration. Therefore, the main research question central for this study is:

What are the lessons learned for achieving cross-sector collaboration, when initiating a project for a healthy living environment?
  - Which strategies to improve cross-sector collaboration were implemented when initiating cross-sector collaboration projects for a healthy environment and to what outcomes?
  - What where the underlying contexts and mechanisms that led to these outcomes?

## 2. Methods

For this study, the realist evaluation (RE) approach was used as the methodological lens for the study’s analysis [20]. RE aims to answer the question what works, for whom, when, and how [20,23]. According to the RE approach, individuals respond differently to interventions within different contexts [24]. Within the RE approach, context–mechanism–outcome (CMO) configurations are used as heuristics to provide insight into why a strategy or resource works in context A but not in context B [24]. In order to provide action-oriented insights into how to successfully collaborate for a healthy living environment, this study focused on understanding which strategies could be implemented within which contexts, to trigger certain mechanisms and, thus, create (preferred) outcomes for cross-sector collaboration for a healthy living environment. We have, therefore, added the concept of strategies (S) to the CMO configuration, thus using SCMO configurations to analyze the data. The strategies are seen as an addition to the CMO configuration; providing more explanatory insight into what actions can be undertaken to improve cross-sector collaboration [25,26]. For the SCMO configurations, the following definitions are based on, and adapted from ([25], Table 1), see Table 1.

### 2.1. Theoretical Framework

In order to gain a deeper understanding of how cross-sector collaboration for a healthy living environment can be achieved, a better understanding of the factors that affect cross-sector collaboration for a healthy environment is needed. The Collaborative Adaptive Health Network (CAHN) Framework had previously been developed in order to understand the components and processes that affect cross-sector collaboration for population health [27]. This framework is based on an international realist synthesis regarding factors that influence complex collaborations for population health (with a corresponding focus on the healthcare sector). The framework consists of eight components and 38 subcomponents, the eight components are social forces, relations, resources, finance, regulations, market, leadership, and accountability [27]. The CAHN framework has been used before as a theoretical framework for understanding collaboration for population health from a wider perspective [25,28]. For this study, we used the CAHN framework as a theoretical framework to help address cross-sector collaboration for a healthy living environment from a wider perspective (including all eight components), while maintaining the possibility of additional factors being put forward based on the collaboration experiences of the three regional projects for a healthy living environment (see Table 2 for the definitions of each of the eight CAHN components).

### 2.2. Setting

This study is part of a larger four-year-long research project, in which cross-sector collaboration processes in regional or local projects for a healthy living environment will be followed throughout their development. This study is based on stakeholders’ experiences of initiating cross-sector collaboration within three regional projects in the Netherlands. These projects were selected because (1) they addressed a variety of themes within policymaking for a healthy living environment, and (2) cross-sector collaboration was required to address the aims of the projects. The variety of challenges addressed within these regional projects (namely, concerns regarding local air quality, effects of climate change on health, and policy priority setting with citizens) was used within this study to provide a broad perspective of experiences related to cross-sector collaboration for a healthy living environment. See Box 1 for more information about the three projects.

### 2.3. Data Collection

Semi-structured interviews were held with the local partners of the three projects described in Box 1. The partners of these projects were included within this study because of their involvement in the initiating phase of the regional projects. In total, seventeen partners of these projects were interviewed, including representatives of citizens (1), farmers (2), the provinces (regional government) (2), municipalities (4), researchers from a national knowledge institute (RIVM) (3), public health services (2), knowledge institutes/universities (2), and representatives of the regional safety services (1), which were the regional partners that were mainly involved in starting up the projects, as mentioned in Box 1. See Supplementary Table S1 for the interviewed partners for each project. The interviews were performed with a semi-structured interview guide. This guide included questions about the partners’ roles and objectives within the projects in order to gain more insight into their reasons for collaboration. Furthermore, the partners were asked about their experiences of collaborating within the projects. Following the RE approach, we specifically asked about the strategies that were used, the contexts in which these were performed, what happened (outcome), and why this happened (mechanisms), thus gaining a deeper understanding of “what works how, why, and when”. In order to ensure a better insight into regional partners’ experiences of cross-sector collaboration, near the end of the interview a printed version of the CAHN framework with visuals of the eight factors for complex collaborations was used as a tool within the interview guide. This tool was meant to help the participants remember other relevant experiences in collaboration that they had not yet mentioned before (see Supplementary S2 for the interview guide).

Box 1 **Description of regional projects.**

**Description of regional projects**

*Project A: Focused on policy priority setting with citizens*

This project addresses a municipality’s wish to include citizens in priority setting for a healthy living environment. Partners included in this project are universities and national knowledge institutes, public health services, municipalities, and a regional safety service. The project has formed a consortium and is being rolled out, after a pilot phase within one municipality, to more municipalities across multiple Dutch provinces. The municipality in which the project has started was focused on within this research.

*Project B: Addressing a discussion about air quality by measuring the air quality together*

The reason for this project was a discussion within a rural municipality, between the local government, citizens, and farmers about the possible effects of intensive livestock farming on air quality and subsequently the (perceived) health of the citizens. There was a need for an independent partner to help address discussions and tensions between the municipality, residents and farmers. Therefore the national knowledge institute was asked to participate in this project by all three parties. A project group was formed with the national knowledge institute, citizen representatives, farmer representatives, civil servants, and a representative from the province. A project plan was agreed upon by the partners. The first steps in carrying out the project plan were taken.

*Project C: Providing a knowledge base for making policy decisions regarding the effects of climate change on health*

This project is based on an existing consortium of knowledge institutes/universities, the regional and local government, and entrepreneurs that had collaborated before. During the time this consortium waited for new opportunities to collaborate, the consortium partners decided to collaborate in a new project with a national knowledge institute. In this project, the partners aim to provide a knowledge base to understand the effects of climate adaptation measurements on health. This project is starting with a needs assessment for this knowledge base among the governmental partners (municipalities and the province).

### 2.4. Data Analysis

The interviews were recorded after informed consent was given. Transcripts were transcribed literally. The interviews were analysed within the MaxQDA 2018 program by two researchers. Coding was based on the realist approach, which means that within each interview the link between context–mechanism–outcome and the performed or intended strategies were searched for. These SCMO configurations were then coded, based on the triggered mechanism, within the components of the CAHN framework, or within a new, additional code. Researcher 1 (N.J.E.v.V.) identified the SCMO configurations and coded them within the CAHN framework, and researcher 2 (E.d.W.) cross-checked these configurations and codes. Differences in configurations or coding were discussed by the two researchers. The final configurations were checked by the research team. No codes additional to the CAHN framework were needed for coding the SCMO configurations. To provide general themes overarching the three projects, the coded SCMO configurations of all interviews were then thematically clustered based on the combination of (successful and unsuccessful) strategies that were implemented and the desired outcomes.

## 3. Results

Based on the interviews with seventeen partners of the three regional projects, seven themes for addressing cross-sector collaboration were identified, namely (1) creating a feeling of equivalence among the partners, (2) building trust among the partners, (3) creating a connection between the different sectors and perspectives, (4) providing clarity about roles and tasks, (5) creating and leveraging reasons to commit to the cross-sector project, (6) making sure the partners feel engaged within the cross-sector project, (7) understanding whom to engage at which point of the process.

**Figure 1:**
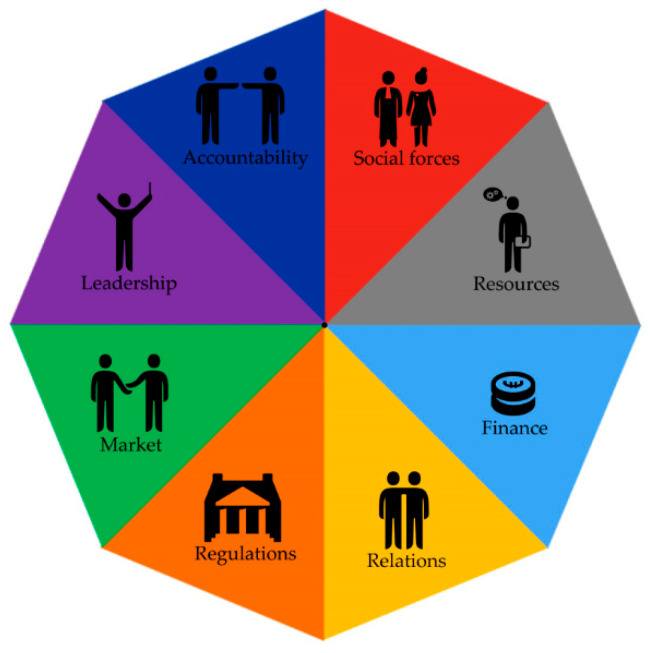
Visualization of Collaborative Adaptive Health Network (CAHN) components.

**Figure 2:**
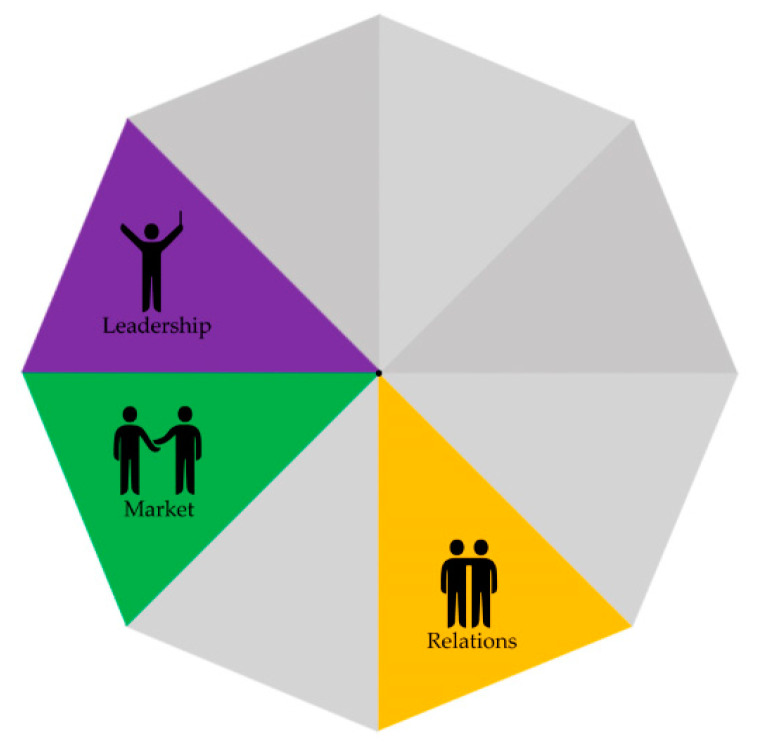
Creating a feeling of equivalence.

**Figure 3:**
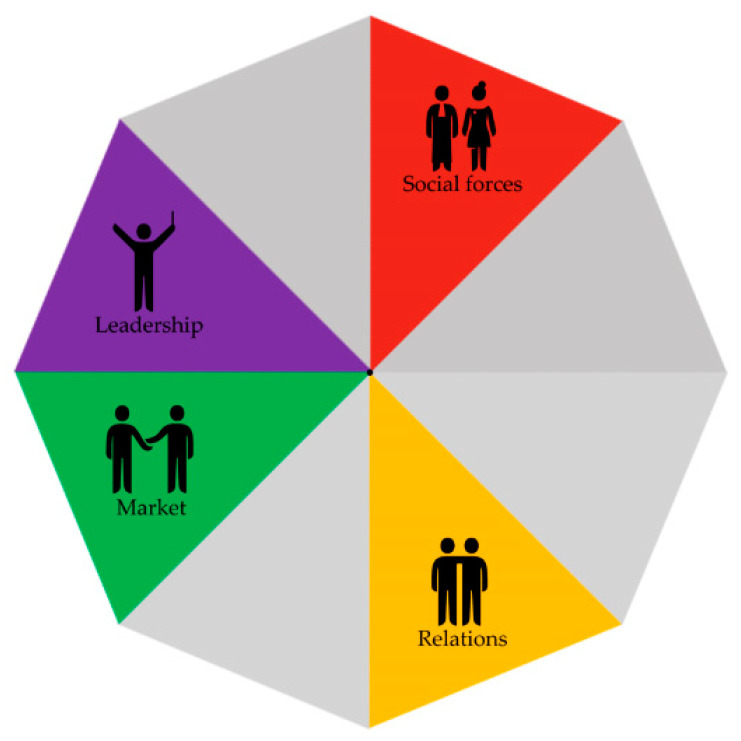
Building mutual trust.

**Figure 4:**
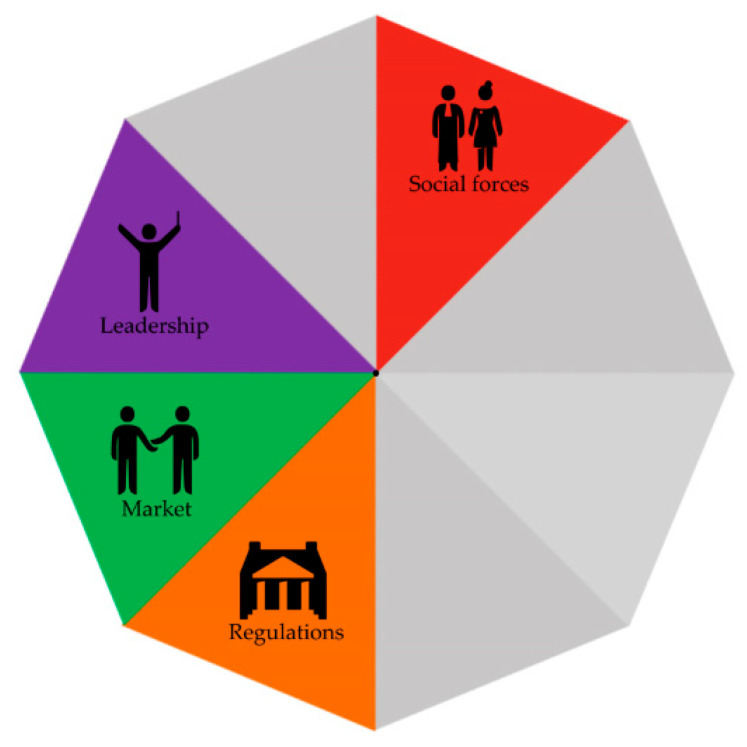
Bridging perspectives.

**Figure 5:**
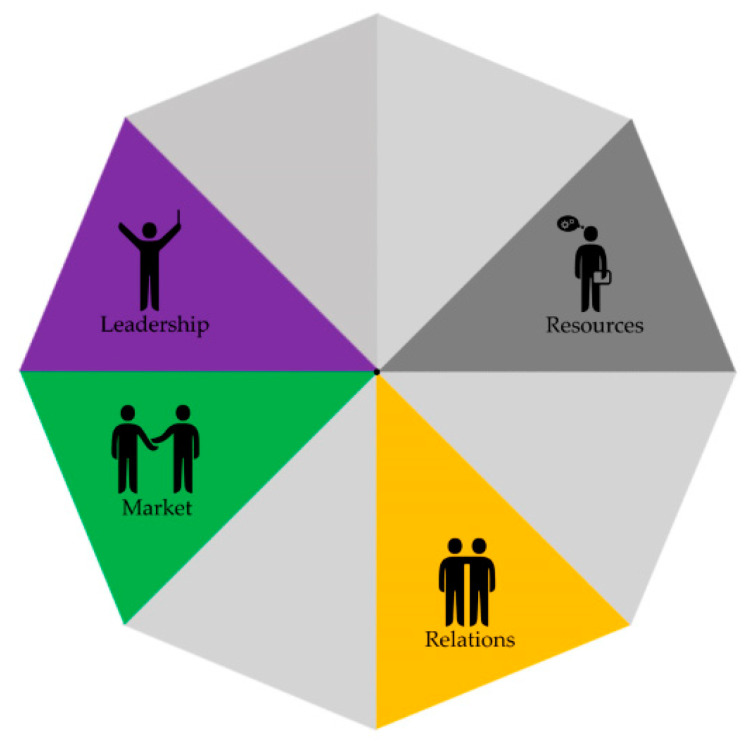
Providing clarity of roles and tasks.

**Figure 6:**
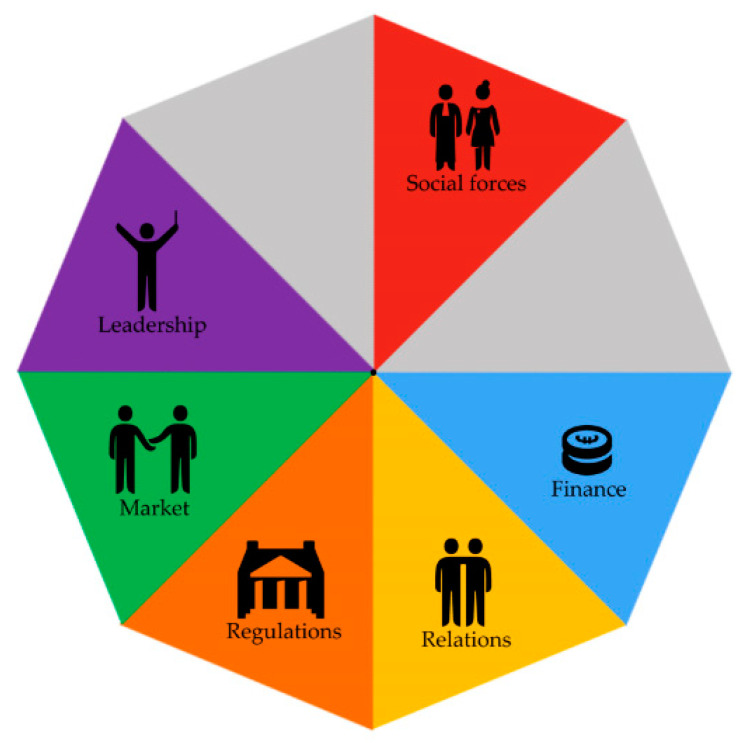
Creating a reason to commit.

**Figure 7:**
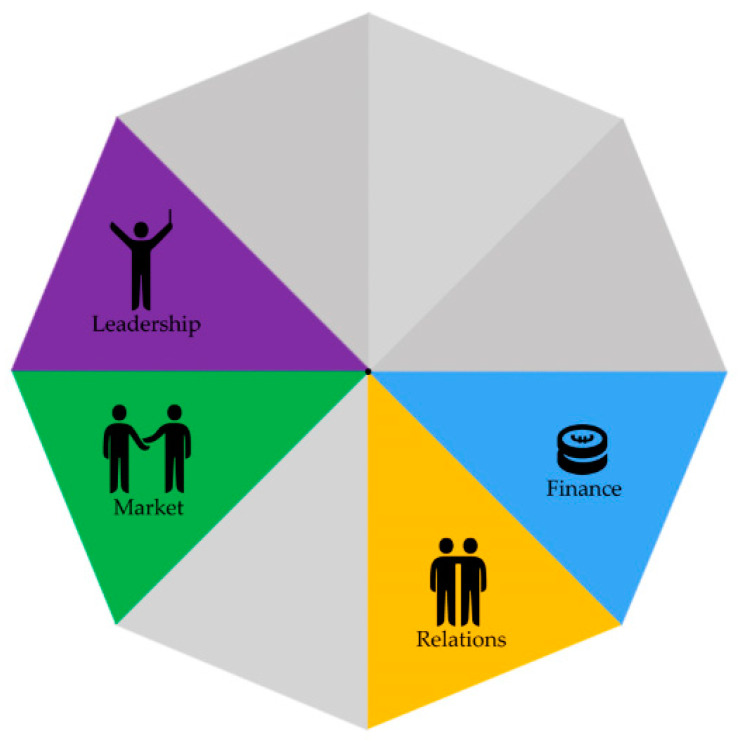
Addressing feeling of engagement.

**Figure 8:**
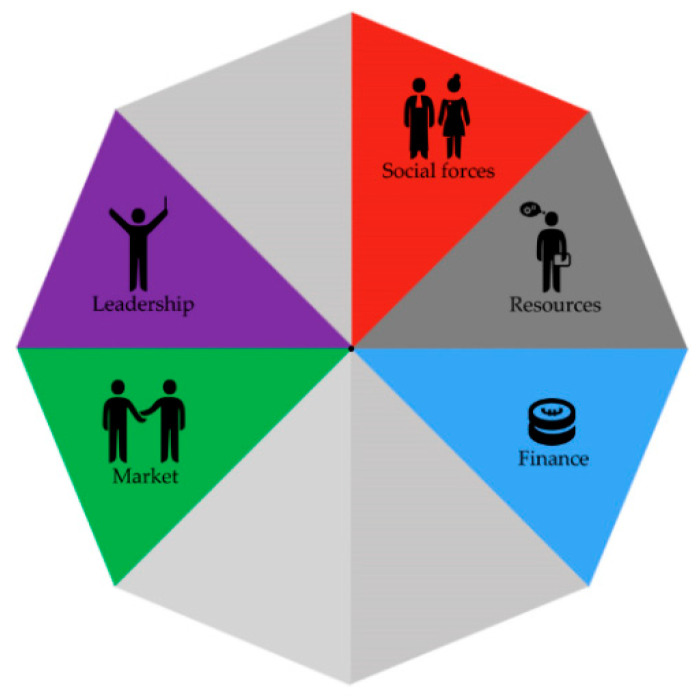
Understanding whom to engage and when.

In the section below, each theme will be addressed, together with a description of the manner in which strategies were implemented within the different contexts and the mechanisms that were consequently triggered (S–C–M–O). After clustering the SCMOs of each theme within the CAHN components, we found different CAHN components were mentioned for addressing the different themes. In order to provide an overview of which CAHN components were addressed within each theme, we have visualized the eight main components of the CAHN framework in Figure 1 and specified the relevance of these components for each theme in Figure 2, Figure 3, Figure 4, Figure 5, Figure 6, Figure 7 and Figure 8.

### 3.1. Creating a Feeling of Equivalence among the Partners

The strategies for addressing a feeling of equivalence among the project partners were mainly mentioned by partners that experienced hierarchical difficulties themselves. These strategies were aimed at addressing a perceived unevenness in the different partners’ expertise or skills, e.g., between national institutes and local institutes, and in contexts where a partner acted upon the perceived hierarchical difference between a professional and a citizen representative (projects A and B). Different strategies were implemented to address this feeling of (in)equivalence, which triggered different mechanisms related to the way in which leadership was used to create a trusting environment, the experienced and acted upon hierarchical or expertise and skill differences between organizations (market), and the ways in which partners communicated with each other (relations) (see Figure 2). See Supplementary Table S3 for more SCMO examples underpinning this theme.

For example, in collaboration with national and local knowledge institutes, a municipal health service experienced a difference in expertise related to research (C). By creating open and regular communication within the project (S), the municipal health service started to understand its own value within the collaboration (M), and due to this feeling of equivalence, they felt more able to speak up and share ideas (O).

*“So, in the beginning, I guess, I felt small, insignificant, until, that relationship improved, and things went more smoothly. And then I thought to myself, hey I do have lots of useful and interesting information to share and I do know the municipality much better.”*

*(R1-I7)*

### 3.2. Building Mutual Trust among the Partners

The need for creating mutual trust was mentioned in all projects as a way to address different perspectives on the problem to be solved or to include new collaboration partners while initiating the project. Strategies in this cluster (see Figure 3) were related to the ways of communicating within the project between partners representing organizations (market) and partners as individuals (relations), bridging the difference in perspectives on the regional problem (social forces), and informing the choice for a neutral organization in a leadership role (leadership).

When collaborating with partners who have different perspectives on a sensitive topic (C), forming an agreement about what is communicated and when (S) helped to create a trusting environment (M) and was experienced as positive by the interviewee (O). In another context where the main focus was to start a new project with different partners (C), building on previous relations (S) provided a basis of trust (M) and stimulated open communication in further collaboration (O).

*“Openness and honesty. So, honoring the commitments. And even if they say ‘all data will stay within the project. There will be no communication about the project before internal agreements are reached about what is communicated’. That is really important. In such a project you need 100% trust within each other.”*

*(R1-I6)*

### 3.3. Creating a Connection between the Different Sectors and Perspectives

Collaboration aimed at a healthy environment involves linking up different sectors and perspectives. This need was mentioned in all three projects and was mainly focused on the lack of proper collaboration within organizations (for example municipalities) and between partners with different perspectives on the issues that were central to the projects. Strategies were based on triggering mechanisms related to the ways of sensemaking of the “problem to address” within the different sectors (social forces) or to the influence of partners’ priorities that were not (yet) focused on integrating perspectives (market). Moreover, the level of committed leadership and representation of all partners’ perspectives within the collaboration (leadership) was mentioned, as were the regulations that discouraged integrated collaboration (regulations) (see Figure 4).

One of the partners mentioned the importance of understanding the effect of the level of collaboration within the separate organizations on the collaboration within the project (S). For example, within the municipality there was insufficient collaboration between the health and environmental policy domains. In addition, due to a recent rehousing, these sectors were now working from different locations (C). This context, in combination with having different priorities within each of the domains, prevented the civil servants of both domains to be triggered to just “walk into each others’ offices” (M). This led to the need for better a connection between both domains as this was perceived necessary for integrating the project within the municipality (O).

This difficulty of connecting both domains within the project was also felt in another project:

*“What I do see is that they [project partners] have difficulty in getting their colleagues [from another domain] to the table. […] What I really appreciated after our meeting with [names project partners which have colleagues from different domains] is that they mentioned afterwards ‘well this was a useful talk, not the least because we now also heard what happens within the other domains [of the organization]’.”*

*(R1-I16)*

### 3.4. Providing Clarity about Roles and Tasks

The need for role clarity was mentioned within all three cases, but especially in case C. Case C was in the phase of figuring out which partners to include, why, and for what objectives. In this context, partners were trying to understand their roles within the project (relations) and searching for an understanding of what to expect from other organizations (market), and were searching for a shared vision for the project (leadership) (see Figure 5). In the other projects, role clarity was mainly mentioned by organizations that were struggling to combine their organizational roles with their role in the project. Strategies that were mentioned by these partners triggered mechanisms related to partners taking up their roles within the project, creating distributed leadership (leadership), the need for better communication (relations), and when they are expected to invest in the project (resources).

For example, the need for role clarity for partners within a project (S) was found relevant in a context where the national knowledge institute collaborated with a regional knowledge partner. When the national knowledge institute took up more work in the region, this was perceived as a role that overlapped with the role of the regional knowledge partner (M), causing distrust (O). Openly discussing the differences between both organizations’ roles and stating that the regional partner would remain the first communication point for the municipality (S) provided more clarity for the regional partner in what to expect (M) and prevented disruption of the collaboration (O).

This last example has been mentioned by a regional partner that “witnessed” the situation:

*“So, it is still the case that the municipality sees the regional partner as first point of contact (...)*

*(Interviewer: And that was mentioned by the municipality?). The municipality verified this in their own words indeed. (…) It gave clearance as it fitted the perceptions the regional partner had about its role (about the effect on the regional partner).”*

*(R1-I12)*

### 3.5. Creating and Leveraging Reasons to Commit to the Cross-Sector Project

During the clustering of the SCMO configurations, a difference between commitment to the project and active engagement in the project was found, and this difference will be explained within this theme and theme six. Creating and leveraging reasons for commitment was mentioned in all three projects and was related to the urgency for partners to collaborate in the projects. Strategies and mechanisms to address this outcome were related to the influence of (perceived) political and societal urgency within the regional project (social forces and regulations), urgency felt by organizations to explore how to address a healthy living environment through regional collaboration (social forces), the inclusion of partners that are thought to have more impact in the collaboration (leadership), and aligning the needs of the organizations with the aims of the regional project (market and finance) (see Figure 6).

One of the partners mentioned that in order to create commitment it was important to have open communication at the start of the project in order to understand the aims, roles, and values of the partner organizations (S). During the first conversation with a possible new regional partner, which often had a financing role in collaboration, the communication was directed at understanding each other’s aims and values. There was no conversation about finances yet (C). According to this partner, this would help create connections between the partners based on their own organizations’ aims and values. The partner mentioned that when the commitment was achieved in these first conversations, financing would be based on the feeling of commitment (M). The first conversations were experienced as valuable and a reason to continue the search for how to collaborate (no definitive collaborative agreements were made during the time of these interviews) (O).

*“Well, if all partners think: yes my needs are included and discussed and I see value [in the project]. We’re all on the same page, so then we all know; ok this is it, this is what we will research and about to produce. And then there’s the moment that we can check, what will be the costs? And then it is easier, because when I see the value, I am willing to pay for it.”*

*(R1-I14)*

### 3.6. Making Sure the Partners Feel Engaged in the Cross-Sector Project

In all three cases, strategies were aimed at creating and maintaining the engagement of project partners, which entails active participation in the project. Creating the feeling of engagement was addressed in two cases by using the right type of leadership (leadership). Depending on the situation, a charismatic leader helped to create engagement, or, in a situation with a lack of mandate, connecting leadership was applied. Also, meetings were organized, and intermediary results were communicated (relations). Finally, in one of the projects the idea was mentioned by one of the partners to let one of the partners have a financial stake in the project to create more urgency to engage (finance) (see Figure 7).

The need to communicate intermediary results to maintain engagement (S) was addressed in a context in which citizens had participated in a priority setting meeting for a healthy living environment (C). A regional partner talked about the citizens investing in this project and felt the need to communicate with them about the results of this project, to show how the citizens’ input was used (M), however due to the long project process, it would take over a year before results could be communicated (O).

*“But I am thinking …like, the citizens appreciate going to a meeting. They are invited to a load of activities, regardless, but well, then they have had their say, and then they would also appreciate seeing what happens with their input. And this shouldn’t take too long. So for a follow-up of the project, shorten the planning. Make sure that it is clear within one year what measures are taken [based on the input of the citizens].”*

*(R1-I10)*

### 3.7. Understanding Whom to Engage at Which Point of the Process

Strategies regarding whom to include in the project were mentioned in all three projects. Choosing to engage new partners can be based on creating a broad expertise within the project (leadership and resources), engaging partners early to increase the chance for (financial) support in the future (finance). However, engaging partners early in the process can also slow down the process, as the needs of more organizations needs to be balanced with project objectives (market and social forces) (see Figure 8).

An example was mentioned about the relevance of engaging a governmental partner early in the project (S). This was based on the expectations that this partner would be needed later in the process but would need some time to engage in the project (C). Involving the partner early in the project enabled the partner to be actively involved in addressing certain problems within the project (M). According to the partner organization that gave this example, problems were still not solved directly, but this helped in addressing problems nonetheless (O).

*“It’s important that a province and a municipality are engaged directly. We can arrange all sorts of things, but if the government is not engaged from the start, before you have organized for them to get engaged, it might take one, two years. By engaging the government from the start and throughout the process, and also showing them the problems we encounter, it is then often easier to get things organized. This does not mean that things are taken care of within half a year. But it will help the government to consider: how can we tackle the problem.”*

*(R1-I6)*

## 4. Discussion

Cross-sector collaboration is found to be complex and context-specific [8,19]. By using the realist evaluation approach, this study identified how seven themes within cross-sector collaboration for a healthy environment were addressed, namely; (1) creating a feeling of equivalence among the partners, (2) building mutual trust among the partners, (3) creating a connection between the different sectors and perspectives, (4) providing clarity about roles and tasks, (5) creating and leveraging reasons to commit to the cross-sector project, (6) making sure the partners feel engaged in the cross-sector project, and (7) understanding whom to engage at which point of the process. By specifying the causal links between strategies, contexts, mechanisms, and outcomes for each of these themes, this is, to our knowledge, the first study to take into account this type of complexity in understanding how cross-sector collaboration for a healthy living environment can be achieved (see Supplementary Table S3 of the total summary of SCMO configurations per theme). The relevance of understanding the influence of context and the triggered mechanisms on how cross-sector collaboration can be achieved was obvious when examining the three projects included in this study. For example, the need for role clarity was mentioned within all three cases, but different strategies were needed, depending on the context of the collaboration. In addition to other studies arguing about the need for role clarity (Mundo et al., 2019), this study provides action-oriented insights as to which strategies can be used best, and why, based on the context of the collaboration.

Most of the themes identified were consistent with previous literature about cross-sector collaboration [7,8,10,19,29]. However, the theme about creating a feeling of equivalence was mentioned in few studies on cross-sector collaboration. The role of power balances between organizations has been mentioned before [30,31]; however the step hereafter, namely how to create a feeling of equivalence, has been mentioned less in cross-sector collaboration literature. As there is a growing call to include more nongovernmental partners in the discussion about a healthy living environment, notably citizens, the need for this feeling of equivalence might be of even more importance when collaborating for a healthy living environment [3,32]. This study provides additional insights into what creating a feeling of equivalence means for cross-sector collaboration and how a feeling of equivalence can be achieved. For example investing in open communication, an understanding the local context, and aiming for distributed leadership can all play a role in creating a feeling of equivalence (see Supplementary Table S3 for detailed SCMO configurations in how to achieve this feeling of equivalence).

In this study, we used the CAHN framework to gain a broader understanding of the components influencing cross-sector collaboration. As can be seen in the results section, according to the experiences within the three projects, different CAHN components were shown to be of relevance within different themes for cross-sector collaboration. We found the factors leadership and market (interorganizational relations) being addressed across all themes. Furthermore, the factors relations (interpersonal relations) and social forces were also addressed in most themes. This is in line with other studies in cross-sector collaboration addressing the relevance of the right type of leadership [8,19]; the relevance of building relations and trust between individuals and organizations [10,30]; and the influence of each partners’ norms, values, and perspectives in the collaboration [30]. While some of the components were mentioned less within the seven themes, (accountability, resources, regulations, and finance) this does not mean that these were not relevant for cross-sector collaboration. The limited representation of SCMO configurations related to these CAHN factors might be related to the phase of the collaboration the three cases were in. All cases recently started their collaborative project and have focused mostly on searching for common objectives and appointing roles and tasks. There was yet little experience with carrying out the project plans. From an earlier study on population health management projects, we found similar results. In the earlier phases of the collaboration, mainly the need for gaining mutual engagement for the project aims, building relations (both between individuals and organizations), and building on leadership were found to be of relevance [25].

### 4.1. Study Limitations

Part of the research team is employed by one of the project partners, the national knowledge institute. Even though the research team was not involved in any of the collaborative projects, we should take into account the possible effect this may have had. In order to prevent bias in the research teams’ perspectives on the collaboration, within the interviews, the national institute was treated the same as the other interviewees, as one of the partners of the collaborative project. Furthermore, the research team explicitly communicated to the other partners that they were not part of the collaborative project and were working independent of the colleagues that were part of the collaborative project.

The framework used for this study was based on a literature study for cross-sector collaboration in population health [27]. The use of this framework can be valued for its broad perspective on cross-sector collaboration, both including process factors and structure factors, a combination valued in cross-sector literature [30]. We have looked at cross-sector collaboration in a healthy living environment, being aware of a possible need for adaptations for the CAHN framework. However, while providing all interviewees the possibilities to address additional factors to the CAHN framework, no additional factors for the framework were mentioned. One participant however mentioned the need to keep in mind whether the CAHN factors, that were perceived more formal/organization-based by the participant, also fit the experiences of citizens as partners. This can be taken into account in further research. As mentioned in the discussion, some of the factors of the CAHN framework were found to be of less relevance in these three projects. This might be because of the phase of development of the collaboration projects. Future research following the projects throughout their process could help provide more insight into the relevance of these CAHN factors for collaborating for a healthy living environment.

### 4.2. Future Research

These experiences were based on the start of a collaborative project. The context of collaboration is expected to change throughout the duration of the projects. Therefore, further research for understanding collaboration throughout these projects is needed. Furthermore, this study is based on three regional projects, providing the context of collaboration. As there are many other forms of collaboration for a healthy living environment (e.g., structurally integrating health in all policies or developing multisector health partnerships, both including various stakeholders with different perspectives), further research including additional contextual differences while collaborating for a healthy living environment is valued.

## 5. Conclusions

Cross-sector collaboration for a healthy living environment is found to be complex and context dependent. This study aimed to provide insight into how this cross-sector collaboration can be achieved by unraveling which strategies can be implemented in which contexts and why. The success for starting cross-sector collaboration projects was largely influenced by the choice of leadership and by interorganizational relations. The context-specific insights from this study can guide regional partners while starting to collaborate in projects for a healthy living environment. Further research broadening these context-specific insights toward other contexts and different collaboration types is valued.

## Figures and Tables

**Table 1:** Conceptualizations of S–C–M–O.

Strategy	Refers to intended plans of action [24]. In this study, the strategies are aimed at achieving cross-sector collaboration for a healthy living environment.
Context	Pertains to the “backdrop” of programs, which can be understood as any condition that triggers or modifies the mechanism [24]. In this study, the contextual conditions can be the different multilevel sociocultural, relational, economic, political, or historical conditions in which the strategies are implemented, which in turn causes certain mechanisms to be triggered.
Mechanism	Refers to the generative force that leads to outcomes [24]. Mechanisms should not be mistaken for strategies, as strategies are seen as intended plans of action, whereas mechanisms are the responses to the intentional resources that are offered [24].
Outcome	Refers to the intended or unintended process outcomes [24]. This study focuses on the outcomes of strategies for achieving cross-sector collaboration for a healthy living environment.

**Table 2:** Definitions of the eight components of the Collaborative Adaptive Health Networks framework (as described in [27]).

Social forces	Social forces anchored at the institutional level consist of three broad types of forces that supply guidelines for the behavior of people: cultural-cognitive (what generally does happen), normative (what should happen), and regulative (what must happen).
Resources	The demand and supply side of resources and the technologies available to organizations, in order for organizations to produce services.
Finance	The management of financial arrangements, which contains three elements: financial strategies, contractual relationships and contractual scope, and requirements.
Relations	How (a new) culture is enacted at the interpersonal level and comprises seven constructs: trust, mindfulness, heedfulness, respectful interaction, group diversity, social and task relatedness, and communication effectiveness.
Regulations	Regulations refers to the national (federal)—state (provincial) and/or county (municipal) health policy and accompanying laws and regulations and to political influence, problem streams, and the political agenda.
Market	The local market refers to four elements that influence the working relationships between organizations within a local health care market (trust–reciprocity–respect; agreement on purpose and needs; engagement; history of the local market) and to the structures and dynamics of this local market.
Leadership	Leadership structures, processes, and styles that provide support and direction for the development of Population Health Management across organizations and sectors.
Accountability	Processes by which one party reports to another on its actions or performance either with or without consequences, i.e., who, what, and how.

## Supplementary Materials

The following are available online at https://www.mdpi.com/1660-4601/17/17/6250/s1. Supplementary Table S1: Interviewed regional partners. Supplementary S2: Interview guide. Supplementary Table S3: Strategy-Context-Mechanism-Outcome configurations per theme for cross-sector collaboration.

## References

[1] Dahlgren G., Whitehead M. (1991). Policies and Strategies to Promote Social Equity in Health.

[2] De Vries S., Verheij R.A., Groenewegen P.P., Spreeuwenberg P. (2003). Natural environments—Healthy environments? An exploratory analysis of the relationship between greenspace and health. Environ. Plan..

[3] Staatsen B.A.M., Alphen T.V., Houweling D.A., Ree J.V.D., Kruize H. (2017). Gezonde Leefomgeving, Gezonde Mensen.

[4] Storm I. (2016). Towards a HiAP Cycle: Health in All Policies as a Practice-Based Improvement Process.

[5] Hendriks A., Jansen M.W.J., Gubbels J.S., Vries N.K.D., Molleman G., Kremers S.P.J. (2015). Local government officials’ views on intersectoral collaboration within their organization—A qualitative exploration. Health Policy Technol..

[6] Spitters H.P.E.M. (2019). In2Action: A Policy Game to Enhance Collaboration in Publichealth in Three European Countries.

[7] Leeuw E.D. (2017). Engagement of sectors other thanhealth in integrated health governance, policy and action. Annu. Rev. Public Health.

[8] Bryson J.M., Crosby B.C., Middleton Stone M. (2006). The design and implementation of cross-sector collaborations: Propositions from the literature. Public Adm. Rev..

[9] Storm I., Hertog F.D., Oers H.V., Schuit A.J. (2016). How to improve collaboration between the public health sector and other policy sectors to reduce health inequalities?—Study in sixteen municipalities in the Netherlands. Int. J. Equity Health.

[10] Mundo W., Manetta P., Fort M.P., Suauaia A. (2019). A qualitative study of health in all policies at the local level. J. Health Care Organ. Provis. Financ..

[11] Molnar A., Renahy E., O’Campo P., Muntaner C., Freiler A., Shankardass K. (2016). Using win-win strategies to implement health in all policies: A cross-case analysis. PLoS ONE.

[12] Taylor-Robinson D.C., Lloyd-Williams F., Orton L., Moonan M., O’Flaherty M., Capewel S. (2012). Barriers to partnership working in public health: A qualitative study. PLoS ONE.

[13] Erickson J., Milstein B., Schafer L., Evans-Pritchard K., Levitz C., Miller C., Cheadle A. Progress along the Pathway for Transforming Regional Health: A Pulse Check on Multi-Sector Partnerships. https://www.communitycommons.org/entities/29c9e981-3167-4311-a851-62ca6c012967.

[14] Burgess T., Braunack-Mayer A., Tooher R., Collins J., O’Keefe M., Skinner S.R., Watson M., Ashmeade H., Proeve C., Marshall H. (2015). Optimizing intersectoral collaboration between health and education: The Health Bridges study. J. Public Health.

[15] Tooher R., Collins J., Braunack-Mayer A., burgess T., Skinner S.R., O’Keefe M., Watson M., Marshall H.S. (2017). Intersectoral collaboration to implement school-based health programmes: Australian perspectives. Health Promot. Int..

[16] Gray B. (1989). Collaborating: Finding Common Ground for Multiparty Problems.

[17] Thomson A.M., Perry J.L. (2006). Collaboration processes: Inside the black box. Public Adm. Rev..

[18] Siegel B., Erickson J., Milstein B., Evans Pritchard K. (2018). Multisector partnerships need further development to fulfill aspirations for transforming regional health and well-being. Health Aff..

[19] Guglielmin M., Muntaner C., O’Campo P., Shankardass K. (2018). A scoping review of th eimplementation fo health in all policies at the local level. Health Policy.

[20] Pawson R., Tilley N. (1997). Realistic Evaluation.

[21] De Leeuw E., Tsouros A.D., Dyakova M., Green G. (2014). Healthy Cities: Promoting Health and Equity—Evidence for Local Policy and Practice.

[22] Shankardass K., Renahy E., Muntaner C., O’Campo P. (2014). Strengthening the implementation of health in all policies: A methodology for realist explanatory case studies. Health Policy Plan..

[23] Wong G., Westhorp G., Manzano A., Greenhalgh J., Jagosh J., Greenhalgh T. (2016). RAMESES II reporting standards for realist evaluation. BMC Med..

[24] Jagosh J., Macaulay A.C., Pluye P., Salsberg J., Bush P.L., Henderson J., Sirett E., Wong G., Cargo M., Herbert C.P. (2012). Uncovering the benefits of participatory research: Implications of a realist review for health research and practice. Millbank Q..

[25] Vooren N.J.E.V., Steenkamer B.M., Baan C.A., Drewes H.W. (2020). Transforming towards sustainable health and wellbeing systems: Eight guiding principles based on the experiences of nine Dutch Population Health Management initiatives. Health Policy.

[26] Weger E.D., Vooren N.J.E.V., Wong G., Dalkin S., Marchal B., Drewes H.W., Baan C.A. (2020). What’s in a realist configuration? Deciding which causal configurations to use, how and why. Int. J. Qual. Res. Methods.

[27] Steenkamer B., Drewes H., Putters K., Oers H.V., Baan C. (2020). Reorganizing and integrating public health, health care, social care and wider public services: A theory-based framework for collaborative adaptive health networks to achieve the triple aim. J. Health Serv. Res. Policy.

[28] Steenkamer B., de Weger E., Drewes H., Putters K., van Oers H., Baan C. (2020). Implementing Population Health Management: An international comparative study. J. Health Organ. Manag..

[29] Ansell C., Gash A. (2007). Collaborative governance in theory and practice. J. Public Adm. Res. Theory.

[30] Bryson J.M., Crosby B.C., Middleton Stone M. (2011). Designing and implementing cross-sector collaborations: Needed and challenging. Public Adm. Rev..

[31] Naaldenberg J., Vaandrager L., Koelen M., Wagemakers A., Saan H., Hoog K.D. (2009). Elaborating on systems thinking in health promotion practice. Global Health Promot..

[32] Weger E.D., Vooren N.J.E.V., Drewes H.W., Luijkx L.G., Baan C.A. (2020). Searching for new community engagement approaches in the Netherlands: A realist qualitative study. BMC Public Health.

