# Supplementary material for: Cross-Sector Collaboration for a Healthy Living Environment—Which Strategies to Implement, Why, and in Which Context?
Source: Int J Environ Res Public Health. 2020 Aug 27;17(17):6250. doi: 10.3390/ijerph17176250 (PMC7504038; doi:10.3390/ijerph17176250)
Supplement: Supplementary file 1 [file ijerph-17-06250-s001.pdf]

Supplementary **S1. Interviewed regional partners**

Representatives of regional partners	Number of interviewees included		
	Case A	Case B	Case C
Citizens		I	
Farmers (Include actual farmers and farmer representatives)		II	
Knowledge institutes/Universities	I		I
Local health authorities	I		I
Municipality	I	II	I
National knowledge institute	I	I	I
Province		I	I
Regional safety services	I		

Part 1: Phase of collaboration [10 min]

1. **Could you explain how and why you are involved in this project [name project]?**
    a. *How and since when are you involved in this project?*
    b. *What is your reason/aim for participating in this project? What is the aim of your organization [if applicable]?*
    c. *What is your role/the role of your organization in the project?*

2. **What is the current situation of the regional project?**
    a. *What is the aim of the project?*
    b. *What is the reason you are working together with other regional partners on this project?*
    c. *Which phase of the project are you in? What have you accomplished already?*

Part 2: Experiences with collaboration [30 min]

3. **What are your experiences with starting up the collaboration within this project?**
    a. What worked positive for collaboration, and what were bottlenecks?
        i. *Could you illustrate this based on your own experiences?*
        ii. *What happened? [S] What was the effect/what was triggered?[M]*
        iii. *Why did this happen at this specific situation/context? [C]*
        iv. *What was the outcome? [O]*
    b. What are, according to you, the prerequisites for a successful start of a project with regional cross-sector collaboration for a healthy living environment?
        i. *Could you illustrate this based on your own experiences?*
        ii. *What happened? [S] What was the effect/what was triggered?[M]*
        iii. *Why did this happen at this specific situation/context? [C]*
        iv. *What was the outcome? [O]*

4. When you look at this A4 [which is a visualization of the eight components and a question mark showing that there might be other components of importance], you can see factors that, according to an international literature review, play a role in complex collaborations. You have already mentioned some of them [name the factors already mentioned].
When you look at this format, are you triggered with other factors that have played a role in starting up the collaboration within the project?
        i. *Could you illustrate this based on your own experiences?*
        ii. *What happened? [S] What was the effect/what was triggered?[M]*
        iii. *Why did this happen at this specific situation/context? [C]*
        iv. *What was the outcome? [O]*

    a. If not yet mentioned [optional];
    In which way did these factors influence the feeling of engagement for collaboration with other regional partners for a healthy living environment?

        i. *Could you illustrate this based on your own experiences?*
        ii. *What happened? [S] What was the effect/what was triggered?[M]*
        iii. *Why did this happen at this specific situation/context? [C]*

[1] This interview guide was translated to English to improve readability for international readers. The actual interview guide was Dutch, the interviews were performed in Dutch.

iv. *What was the outcome? [O]*

b. If not yet mentioned [optional];
In which way did these factors influence the quality of collaboration?
   i. *Could you illustrate this based on your own experiences?*
   ii. *What happened? [S] What was the effect/what was triggered?[M]*
   iii. *Why did this happen at this specific situation/context? [C]*
   iv. *What was the outcome? [O]*

Part 3: Expectations for collaboration [5 min]

5. **What are your expectations for the next 1,5 years of the project?**
   a. What are possible opportunities?
   b. What are possible threats?

Part 4: Final questions – Role of RIVM and recommendations
One of the aims of this research is to use the knowledge about regional cross-sector collaboration for learning with our regional partner and the RIVM colleagues.

6. **What is the role of the RIVM in this collaboration project?**

7. **What are your recommendations concerning the role of the RIVM in this collaboration project?**

Supplementary **S3: Strategy-Context-Mechanism-Outcome configurations per theme for cross-sector collaboration**[2]

Theme 1: Creating a feeling of equivalence among the partners

**Strategy**	**Context**	**Mechanism**	**Outcome**	**CAHN component**
Understanding the importance of the local context and local language	Cross-sector collaboration with national and local partners. The national partner is a knowledge institute, known from its scientific expertise. The local partner has a combination of scientific and practical expertise.	When the national partner does not understand the language of the local context and communicates solely from a national point of view, in a scientific manner, local partners that communicate in a less scientific manner feel less equivalent.	The national partner can be perceived as arrogant and the communication remains a one-way thing.	Relations<br>*Communication*
Avoiding hierarchical behavior/ engaging self-confident citizen representatives in partnerships with professionals	A cross-sector project in which professionals and citizens collaborate, and the professional portrays himself on a hierarchically higher level	Due to the self-consciousness of the citizen, this behavior causes a reaction to 'shake him of his statue', through which the citizen does not feel less equivalent	Due to the behavior of the citizen the focus is maintained on the shared aim, searching for a way of collaboration in their project	Relations<br>*Respectful interaction*
Using open communication to stimulate collaboration of all partners	A cross-sector project with partners that experience a difference in knowledge or expertise	By using an open and approachable way of communication (regular physical or mail contact, asking advice from all partners) other partners learn about their value on the project and a feeling of equivalence is created.	This causes the more hesitant partners to communicate about their ideas as well	Market<br>*Trust-reciprocity-respect*<br><br>Relations<br>*Communication*
Investing in distributed leadership	A cross-sector project in which multiple partners from different sectors collaborate and a difference in knowledge and expertise is experienced	There is one leader, but all partners take their role and create a feeling of distributed leadership. Even when a partner feels like having a little role, he still feels a level of equivalence as the project is from all of them.	This makes the partner feel relevant within the project, regardless of the role it has.	Leadership<br>*Distributed leadership*

[2]Per theme, Strategies, implemented in the Contexts, triggering Mechanisms that cause certain Outcomes (SCMO) are described. For each SCMO configuration the relevant CAHN components are identified.

Theme 2: Building mutual trust among the partners

**Strategy**	**Context**	**Mechanism**	**Outcome**	**CAHN component**
Creating mutual agreements at the start of the project	A project in which the (intermediary) results of the project can have different effects on different partners, which required clear agreements on how to communicate these results	The creation of mutual agreements on the communication of these results (e.g. first intern communication) created trust among the partners and stimulated open communication between the partners	Despite the possible sensitivity of the results, collaboration at the moment of the interview was experienced as positive.	Market<br>*Trust-reciprocity-respect*
Using an independent 'leader' for creating trust	A project in which partners have different perspectives and stakes regarding the problem that needs to be solved.	also Including an independent 'leader' in charge of the results, creates trust in the results of the project being handled correctly.	Both partners with different perspectives were willing to collaborate in one project, searching for a mutual solution.	Leadership<br>*Representation*<br><br>Social forces<br>*Sensemaking*
Utilizing earlier collaborative experiences for current collaboration	A context in which individuals have worked together before, with positive experiences	As persons know each other from earlier collaborations, they find it easier to communicate in the new project	This is experienced as a positive factor for further collaboration	Relations<br>*History*
Showing appreciation for the local context	A project in which multiple local partners and one national partner collaborate	As the national partner attends multiple local meetings, the local partner gains trust in the willingness of the national partner to really understand the local context	This is experienced as a positive factor for further collaboration	Relations<br>*Trust*
Communicating openly with the partners about changes in the project	A project in which some partners have advocated for their problems for years. This problem was not solved and current project provides new hope.	Not communicating a changed planning of the project by the project leader, created distrust among the partners, as they felt the solution to their problem was postponed again	The project leader then communicated with these partners, which helped maintain their participation in the project	*Market*<br>*Trust-reciprocity-respect*

Theme 3: Create a connection between the different sectors and perspectives

Strategy	Context	Mechanism	Outcome	CAHN component
Using an independent 'leader' in projects with partners having different perspectives	A project with multiple local partners that have various local stakes. The national partner is perceived to have less local stakes, and is perceived more independent about this topic.	In this context the independent partner had also a connecting role during discussions between local partners, being able to neutrally point out the value of each of these partners	According to feedback rounds this role of the national partner was appreciated by the local partners	Leadership<br>*Representation*
Keeping in mind the level of collaboration between sectors within one organization	Within the municipalities health and the living environment are different sectors, between which there is little collaboration (to the level of 'sending each other their documents'). Within one municipality there was a reorganization causing these sectors to be physically distant as well. For the project input of both sectors was needed.	This physical distance, in combination with different priorities with the sectors, causes both sectors to not easily 'walk in' with each other.	Connecting both sectors within the municipality is perceived as necessary for integrating the project within the municipality.	Market<br>*Local market structure*<br><br>Social forces<br>*Mental models*
Organize meetings to bring partners from different sectors together	Collaboration within and between organisations is required for the project, but not achieved yet. Regulations for collaboration and different priorities are mentioned as reasons for not collaborating.	Organizing meetings to bring the partners from different sectors together provides the possibility to address the problem openly during the meeting and shows the partners the value of collaborating	Partners were happy they were able to find each other better when needed	Social forces<br>*Mental models*<br><br>Regulations<br>*Influences of policy*

Theme 4: Providing clarity about roles and tasks

**Strategy**	**Context**	**Mechanism**	**Outcome**	**CAHN component**
Keep the local context into account	A national knowledge institute collaborated in a regional project with a knowledge partner that was regionally focused. When the national knowledge institute started to work more in the region, this was perceived as a role that was overlapping with the original role of the regional knowledge partner, causing distrust.	By clear communication of the difference between both organizations' roles and by stating the regional partner would remain the first communication point for the municipality, this provided clarity for the regional partner in what to expect.	This prevented disruption of the collaboration.	Relations<br>*Heedfulness*<br><br>Market<br>*History of local market*
Creating understanding of the organizations' role within the project and within the organization	A local partner which perceives itself being an executive partners, is included in the meetings of the larger consortium	The organization role and the role within the project do not fit according to the partner, which causes difficulties in combining both roles, especially as there is limited time available.	The partner addresses the need for a broader representative utility taking the project role.	Relations<br>*Heedfulness*<br><br>Resources<br>*Supply side*
Handle expectation management to the stakeholder with the whole project team	In a project with different perspectives on the problem at hand. An independent organization was asked to help find a solution. However, the role of this organization was of a scientific kind, the organization would not be involved in the policy addressing the solution. The stakeholders of the partner organizations expected the more of the independent organization than was possible.	By communicating about the role of the independent partner by multiple partners of the project team, stakeholders gain an understanding of what this role exactly includes within the project	Now partners of the project team actively communicate this role themselves.	Leadership<br>*Distributed leadership*

Theme 5: Creating and leveraging reasons to commit to the cross-sector project

**Strategy**	**Context**	**Mechanism**	**Outcome**	**CAHN component**
Understanding the influence of society and politics on the sense of urgency to engage in the project	There is a societal and political interest regarding the healthy living environment.	Due to societal and political influences the partners feel a higher sense of urgency to participate in the project addressing this theme	Participation of partners and priority for addressing this project.	Regulations<br>*Problem stream*<br><br>Social forces<br>*Sensemaking*
Including partners that provide trust and help creating engagement for the project	Within the local project a national partner is participating. The national partner is perceived to bring expertise to the project.	The inclusion of this national partner can create a way for new partners to participate, as this can create a higher feeling of relevance of the project	Expectations for this project were that without the national partner the project wouldn't exist the way it does now.	Leadership<br>*Representation*
Addressing the needs of the partner to create engagement for the project	Within a project a partner had recently experienced organizational changes, causing it to have different priorities and little time and resources left to participate in a new project	By connecting the project aim with the organizational aims, a win-win is searched for, lowering the perceived costs of collaboration	This creates an urgency for the partner to collaborate in the project	Market<br>*Engagement*
Utilizing local partners for connecting with new local partners	A national partner working in a local context wants to engage more local partners in the project. Connection is created via another local partner, which addressed to to have better understanding of the local context and partners.	As the local partner already has a relation with the other local partner (both on personal and organizational level), it feels it is easier to create engagement for the project	The new local partner has decided to participate in the project.	Market<br>*History of local market*<br><br>Relations<br>*History*
Adjust communication to the needs of the partner	Within a project the aim was to engage a municipality, which is often viewed as a financial partner. During the first meeting, the focus was on the mutual interests, not on the finances.	The municipality mentioned the relevance of first creating a mutual understanding of shared interest. Stating that finances would eventually follow if enough urgency is felt.	The meeting was perceived valuable and a start of further collaboration	Market<br>*Engagement*

Theme 6: Making sure the feel engaged to the cross-sector project

**Strategy**	**Context**	**Mechanism**	**Outcome**	**CAHN component**
Using the right skills for leadership in different contexts	There is an independent leader in a project, lacking mandate. Focusing on connecting the partners<br><br>Within a project with different partners there is a charismatic person taking a leadership role.	This requires a leader how is able to create a sense of urgency without other partners realizing it (being more sensitive about it).<br><br>By the charismatic traits of the 'leader' in combination with him listening to the partners an being transparent creates commitment among the partners, not just with the project, but with the person	The lack of mandate can have an effect on the speed of the process of collaboration<br><br>Partners attend meetings not only for the project, but because of the commitment to the person	Leadership<br>*Committed leadership*
Investing in face-to-face meetings	A project with multiple partners who have a different level of involvement in the project.	By investing in face-to-face meetings (bilateral or festive group meetings), the contacts with all partners are maintained and the partners get a feeling they are part of the project	This is mentioned to be relevant for keeping the partners committed to the project	Relations<br>*Communication*
Having partner putting in their resources can help creating commitment	In the project, part of the equipment needed is paid for by a certain partner group	The representative of this partner group mentions the effect of putting in own resources on creating more active commitment, as their investment is at stake.	The representative however also addresses the need for a reimbursement valuing this commitment	Finance<br>*Financial strategy*
Creating commitment by going through a goal defining process together	In a project with partner from different sectors, partners went through a communal process of defining their aims and needs/ writing a research call	By going through this process together, more understanding of the partners' interests and commitment is created	New partners didn't go through this process and feel not as commitment 'as if they are hanging on the side'.	Market<br>*Engagement*

Theme 7: Understanding whom to engage at which point of the process

**Strategy**	**Context**	**Mechanism**	**Outcome**	**CAHN component**
Engaging partners with the aim to create a broad perspective on the problem	A project focusing on addressing a problem from multiple sectors; health and the living environment	By making sure partners from different sectors, having different perspectives, are engaged, there is expertise to broaden the discussion about how to address the problem of the project	This can include partners from multiple organizations, as long as the perspectives of multiple sectors are included.	Leadership<br>*Representation*<br><br>Resources<br>*Supply side*
Weighing project needs with organizational needs	Some project partners need long term input for arranging their organizations as needed for the projects. However, the base of the project is focused on handling the problem in a flexible manner.	This causes a situation in which the project needs and organizational needs are weighed	No decision has been made yet. However for the partners to perform their tasks, they need input in an earlier phase.	Market<br>*Agreement on purpose & needs*
Understanding the time it takes for engaging new partners	In a project the government partners are engaged early in the process, as it is expected that they will be needed later in the project (e.g. for financial reasons)	Previous experiences with including these partners created the expectations that it would take a long time to engage these partners when done during the project when action of these partners is needed	Having this partner engaged helps them already thinking about what they can do for the project	Leadership<br>*Representation*<br><br>Finance<br>*Financial strategy*
